# *Plasmodium yoelii* Erythrocyte Binding Like Protein Interacts With Basigin, an Erythrocyte Surface Protein

**DOI:** 10.3389/fcimb.2021.656620

**Published:** 2021-04-14

**Authors:** Takaaki Yuguchi, Bernard N. Kanoi, Hikaru Nagaoka, Toyokazu Miura, Daisuke Ito, Hiroyuki Takeda, Takafumi Tsuboi, Eizo Takashima, Hitoshi Otsuki

**Affiliations:** ^1^ Division of Malaria Research, Proteo-Science Center, Ehime University, Matsuyama, Japan; ^2^ Division of Medical Zoology, Department of Microbiology and Immunology, Faculty of Medicine, Tottori University, Yonago, Japan; ^3^ Division of Proteo-Drug-Discovery Sciences, Proteo-Science Center, Ehime University, Matsuyama, Japan

**Keywords:** *Plasmodium yoelii*, PyEBL, basigin, invasion, protein-protein interaction, CD147, EMMPRIN

## Abstract

Erythrocyte recognition and invasion is critical for the intra-erythrocytic development of *Plasmodium* spp. parasites. The multistep invasion process involves specific interactions between parasite ligands and erythrocyte receptors. Erythrocyte-binding-like (EBL) proteins, type I integral transmembrane proteins released from the merozoite micronemes, are known to play an important role in the initiation and formation of tight junctions between the apical end of the merozoite and the erythrocyte surface. In *Plasmodium yoelii* EBL (PyEBL), a single amino acid substitution in the putative Duffy binding domain dramatically changes parasite growth rate and virulence. This suggests that PyEBL is important for modulating the virulence of *P. yoelii* parasites. Based on these observations, we sought to elucidate the receptor of PyEBL that mediates its role as an invasion ligand. Using the eukaryotic wheat germ cell-free system, we systematically developed and screened a library of mouse erythrocyte proteins against native PyEBL using AlphaScreen technology. We report that PyEBL specifically interacts with basigin, an erythrocyte surface protein. We further confirmed that the N-terminal cysteine-rich Duffy binding-like region (EBL region 2), is responsible for the interaction, and that the binding is not affected by the C351Y mutation, which was previously shown to modulate virulence of *P. yoelii*. The identification of basigin as the putative PyEBL receptor offers new insights into the role of this molecule and provides an important base for in-depth studies towards developing novel interventions against malaria.

## Introduction

Malaria, caused by *Plasmodium* spp., is a serious infectious diseases of global importance. Understanding how the parasites infect and propagate within host cells as well as how they induce pathological effects is important in the development of effective intervention approaches. *Plasmodium yoelii*, a rodent malaria parasite species, has been widely studied to understand the interactions between malaria parasites and host cells. Three *P. yoelii* parasite lines that differ greatly in their virulence; 17X (non-lethal), 17XL and YM (lethal), have been used extensively to study the molecular and genetic bases of growth rate differences between parasites (Yoeli et al., 1975). *Plasmodium yoelii* 17X mainly infects immature erythrocytes (reticulocytes), while the 17XL and YM lines can infect both mature and immature erythrocytes (Yoeli et al., 1975). Such differences in host erythrocyte invasion preference may be driven by parasite exploitation of different erythrocyte surface receptors. Indeed, a single amino acid substitution in the *P. yoelii* erythrocyte binding-like proteins (EBL) C-terminal Cys-rich domain (region 6) is associated with parasite virulence as well as determining localization of the protein to the micronemes (in the reticulocyte restricted17X line), or to the dense granules (in the non-restricted 17XL line) (Otsuki et al., 2009).

The importance of the EBL proteins in erythrocyte invasion has been demonstrated in several *Plasmodium* species. Identification of the erythrocyte receptors of EBL in each parasite species will contribute to the understanding of the molecular mechanism of erythrocyte invasion. These interactions are important for host-pathogen recognition, erythrocyte invasion and malaria pathology, and are therefore attractive targets for vaccine and/or small molecule inhibitors. *Plasmodium vivax* is highly dependent on its EBL orthologue, the Duffy binding protein (PvDBP) and its receptor on the surface of reticulocytes, Atypical Chemokine Receptor 1 (previously referred to as Duffy antigen receptor for chemokines (DARC)) to infect humans (Akter et al., 2019). Individuals lacking Duffy antigen have been observed to have a lower risk to *P. vivax* infection (Howes et al., 2011). Furthermore, naturally acquired antibodies that block the DBP-DARC interaction, can inhibit erythrocyte invasion by *P. vivax* and are associated with clinical protection (King et al., 2008; Nicolete et al., 2016). PvDBP region 2, the N-terminal cysteine-rich region (Adams et al., 1992) which serves as a ligand to DARC, based candidate vaccine elicits strain transcending functional antibodies in humans (Singh et al., 2018). In *P. falciparum*, EBL orthologues, EBA-175 and EBA-140 that interact with Glycophorin A and Glycophorin C, respectively, have been targeted for vaccine development (El Sahly et al., 2010).

Unlike other EBLs, PyEBL lacks paralogues in the parasite making it an ideal model for understanding how the parasite exploits the molecule during invasion. Although the erythrocyte receptors of PyEBL are believed to be important for erythrocyte invasion, they remain understudied. In addition, the factors that enable mutant 17XL and YM parasites to invade a larger repertoire of erythrocytes than the 17X parasites are not completely understood. In this study, we aimed at systematically identifying the potential receptors for PyEBL on the surface of mouse erythrocytes leveraging the wheat germ cell-free system (WGCFS). Specifically, we developed and screened a library of mouse erythrocyte recombinant proteins against native PyEBL using an amplified luminescent proximity homogeneous assay (AlphaScreen). As a result, we identified mouse basigin, an erythrocyte surface protein, as the putative receptor of PyEBL.

## Materials and Methods

### Production of Recombinant *P. yoelii* and Mouse Erythrocyte Proteins

PyEBL (PY17X_1337400) regions were prepared as described (Otsuki et al., 2009). Briefly, the sequences encompassing the ectodomain or other specific regions and/or domains were amplified from *P. yoelii* 17X genomic DNA by PCR by using sense primers with XhoI restriction sites and antisense primers with NotI restriction sites (**Supplementary Table S1**). The amplified fragments were then restricted and ligated into the pEU-E01-GST expression vector (CellFree Science, Matsuyama, Japan) followed by sequencing with an ABI PRISM 3100-Avant genetic analyzer (Applied Biosystems, Foster City, CA). The GST-tagged proteins were expressed with WGCFS (CellFree Sciences**)** and purified using glutathione-Sepharose 4B columns (GE Healthcare, Camarillo, CA) as previously described (Otsuki et al., 2009).

Expression constructs of putative mouse erythrocyte surface proteins were prepared as previously described (Miura et al., 2018). Membrane and GPI anchored proteins (n=237) were selected from the FANTOM (functional annotation of mouse cDNA) database on the basis of being putative mouse orthologs of the human genes that encode erythrocyte proteins as annotated in the Ensemble (http://asia.ensembl.org/) database (**Supplementary Table S2**). The proteins were expressed using the WGCFS as N-terminal mono-biotinylated recombinant proteins as previously described (Miura et al., 2018). For further characterization, cDNA of the C57BL/6J mouse basigin gene (FANTOM ID: 0610008G13) was ligated into pEU-E01-GST, and protein was expressed with WGCFS and purified using glutathione-Sepharose 4B column (GE Healthcare) as described (Ito et al., 2013). All specific primers used in this study are presented in **Supplementary Table S1**.

### Preparation of *P. yoelii* Parasite Lysate From Infected Mice

*Plasmodium yoelii* parasite soluble extracts were prepared following intraperitoneal injection of 8-week-old female BALB/c mice (Charles River, Yokohama, Japan) with *P. yoelii* 17XL parasites as previously described (Rungruang et al., 2005). Briefly, blood from infected mice was depleted of leukocytes using a CF11 cellulose (GE Healthcare, Buckinghamshire, UK) column. *Plasmodium yoelii* extracts containing PyEBL were prepared by freeze-thawing schizont-infected erythrocyte pellets in the presence of protease inhibitors (1 μg/ml of leupeptin, 1 μg/ml of pepstatin A, 100 μM 4-(2-aminoethyl) benzenesulfonyl fluoride hydrochloride) with 1 mM EDTA. The supernatant containing the soluble extract of approximately 1 × 10^7^ parasites/μl was obtained by centrifugation at 21,600 × g for 10 min and subsequently used for screening assays.

### AlphaScreen Assays

Interactions between PyEBL and the library of mouse erythrocyte proteins was assessed *via* AlphaScreen (PerkinElmer, Waltham, MA) as previously described (Miura et al., 2018). Briefly, the reaction mixture consisted of 1 μl of *P. yoelii* extracts, 1.3 μg/ml mouse anti-PyEBL polyclonal antibodies and 1 mg/ml bovine serum albumin (BSA) (Wako, Osaka, Japan) in PBS, for a total volume of 9 μl/well in a 384-well OptiPlate (PerkinElmer). Biotinylated recombinant mouse protein (1 μl) was then added and incubated at 26°C for 30 min. A mixture of streptavidin-coated donor-beads and protein G conjugated acceptor-beads (PerkinElmer) was added to the mixture and incubated for 1 h at 26 °C in the dark to allow the donor- and acceptor-beads to optimally bind to biotin and IgG, respectively. Upon illumination of this complex, a luminescence signal at 620 nm termed ‘raw AlphaScreen Counts’ (ASC) was detected and quantified by an EnVision plate reader (PerkinElmer). Flotillin 2 (FANTOM ID: 4933417M14) or Duffy blood group (FANTOM ID: 2510001J03) that reacts with anti-Pys25 monoclonal antibody #16 (mAb#16) (Tsuboi et al., 1997) was used as a negative control. After the initial down-selection of the top 18 reactive erythrocyte proteins, the assays were repeated twice including 5 additional GPI-anchored proteins. The standardized magnitude of signal increase (AS Signal) between individual mouse target proteins and the negative control was determined using the formula: (ASC - background ASC (ASC of negative control; NC)/ASC of NC to give the AS Signal.

### Surface Plasmon Resonance

SPR experiments were performed using a Biacore X100 instrument (GE Healthcare) according to manufacturer’s instructions and as previously described (Nagaoka et al., 2019). Briefly, recombinant mouse basigin was immobilized to a CM5 chip (GE Healthcare) by amine coupling. The buffer HBS-EP+ (10 mM HEPES, pH 7.4, 150 mM NaCl, 3 mM EDTA, 0.05% (v/v) surfactant P20) was used as the running buffer for all SPR experiments. Blank flow cells were used to subtract buffer effects on sensorgrams. Recombinant PyEBL regions were assayed as analytes. After subtraction of the contribution of bulk refractive index and nonspecific interactions with the CM5 chip surface, protein-protein association (*k*_a_) and dissociation (*k*_d_) rate constants were obtained by global fitting of the data in a 1:1 binding model equation. Measurement conditions were optimized so that the contribution of mass transport to the observed values of *K*_D_ was negligible.

### Ethics Approval

Animal experimental and immunization protocols were approved by the Institutional Animal Care and Use Committee of Ehime University and the experiments were conducted in accordance with the Ethical Guidelines for Animal Experiments of Ehime University (Ref: SU-17-1).

## Results

### *Plasmodium yoelii* Native PyEBL Interacts With Basigin

**Figure 1:**
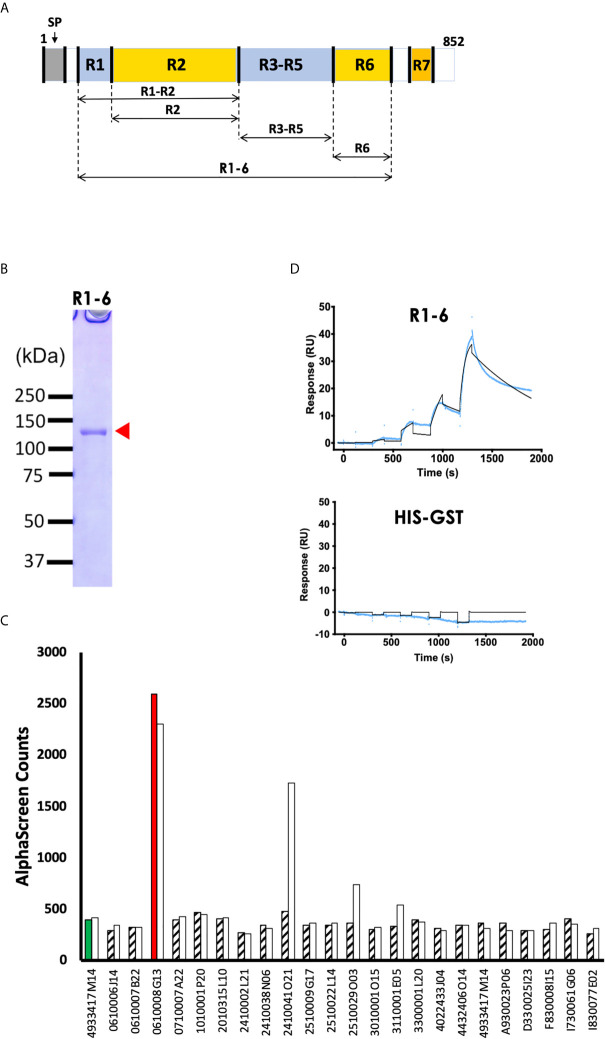
PyEBL interacts with basigin, a mouse erythrocyte surface protein. **(A)** Schematic representation of PyEBL (PY17X_1337400) domain architecture. The protein has a predicted signal peptide (SP; 1 to 22 aa), followed by 7 homology regions (R) 1-7. R2 is referred to as the Duffy binding-like domain; R6 is the C-terminal Cysteine-rich domain. Recombinant PyEBL R1-6 amino acids V_28_-N_788_ as well as truncates corresponding to R1-2, amino acid V_28_-G_437_; R2, E_113_-G_437_; R3-5, G_437_-C_717_; and R6, C_717_-N_788_ were expressed as N-terminal GST-tagged proteins by wheat germ cell-free system (WGCFS). **(B)** WGCFS expressed recombinant PyEBL R1-6 was purified by glutathione-Sepharose 4B column and resolved by 12.5% SDS-PAGE under reducing condition and stained with Coomassie brilliant blue (CBB). The protein resolved as a single band as shown (arrowhead) at the expected molecular weight. All blue ™ molecular marker points are shown. **(C)** AlphaScreen reactivity profile of recombinant PyEBL R1-6 and mono-biotinylated recombinant putative mouse erythrocyte surface proteins. PyEBL R1-6 was mixed with mouse anti- PyEBL R1-6 polyclonal antibodies, 1 μl *P. yoelli* parasite lysate and each of the 237 biotinylated erythrocyte surface proteins and incubated for 1h at 26°C to form an antibody-native PyEBL-mouse protein complex. A suspension of streptavidin-coated donor-beads and protein G conjugated acceptor-beads mixture was then added to the reaction followed by a 1 h at 26°C incubation. This allowed the donor- and acceptor-beads to optimally bind to biotin and rabbit IgG, respectively. Upon illumination of this complex, a luminescence signal at 620 nm was detected by the EnVision plate reader (PerkinElmer) and the result was expressed as AlphaScreen counts (ASC). The top 23 mouse proteins were assayed two times which are represented by a white and a filled pattern bar per protein. The number on the x-axis represent FANTOM IDs. Flotillin 2 (4933417M14) reacted with anti-Pys25 mAb#16 was used as the negative control. Basigin (ID: 0610008G13) had the highest signal. Detailed description is provided in **Supplementary Table S4**. **(D)** Sensorgram of SPR single-cycle kinetic analysis between recombinant PyEBL R1-6 and recombinant mouse basigin. GST tagged ecto-basigin, expressed as illustrated, was immobilized on CM5 chip and used as the ligand while recombinant PyEBL R1-6 was used as analyte. His-GST was assayed as a negative control. Blue curve represents the actual data-generated sensorgram while black curve indicates line of fit used to calculate kinetics parameters. The assay was performed at an increasing analyte concentration of 6, 12, 24, 48, and 96 nM at 120s and dissociation time of 180s. The last dissociation time was extended to 600s to accurately determine kinetic parameters.

Since EBL proteins play an important role in initiation and formation of the tight junction between the apical end of the merozoite and erythrocyte surface, we sought to determine whether PyEBL binds directly to mouse erythrocytes proteins. First, a recombinant PyEBL ectodomain spanning region (R) 1 to R6 (PyEBL R1-6; amino acids V_28_-N_788_) was expressed using WGCFS (**Figure 1A**). Expression was confirmed by SDS-PAGE where it resolved as a single band of 120 kDa (**Figure 1B**). Mouse polyclonal antibodies raised against the recombinant PyEBL specifically recognized the native parasite protein as reported previously (Otsuki et al., 2009).

To identify potential receptors for PyEBL on the surface of host erythrocytes, we conducted AlphaScreen assays with the mouse erythrocyte proteins as previously described (Miura et al., 2018). A total of 237 membrane and GPI anchored mouse orthologs of the human genes that encode erythrocyte proteins were successfully expressed using the WGCFS as N-terminal mono-biotinylated recombinant proteins (**Supplementary Table S2**). The AlphaScreen assay was designed such that the polyclonal antibody binds with native parasite PyEBL which in turn would bind with a recombinant erythrocyte protein (**Supplementary Figure S1**). After the initial down-selection of the top 18 reactive erythrocyte proteins (**Supplementary Table S3**), the assays were repeated twice, this time including 5 GPI-anchored proteins and a negative control. Basigin (FANTOM ID: 0610008G13) had the highest and distinct signal among the 23 erythrocyte proteins, suggesting its interaction with parasite native PyEBL (**Figure 1C**; **Supplementary Table S4**).

To validate the interaction between PyEBL and basigin, we analyzed the strength of the interaction between the two proteins using SPR. We observed that recombinant PyEBL at increasing concentration of 6, 12, 24, 48, and 96 nM, directly interacts with recombinant GST-tagged basigin with an equilibrium binding constant (*K*_D_) of 3.3 ×10^-8^ M (**Figure 1D**). As a negative control, His-GST did not show specific interactions with basigin (**Figure 1D**). Other parameters determined by the SPR are shown in **Supplementary Table S5**. These observations suggest that basigin is the erythrocyte receptor for native PyEBL protein.

### Region 2 Is Responsible for PyEBL Interaction With Basigin, and the Binding Is Not Affected by the C351Y Mutation

**Figure 2:**
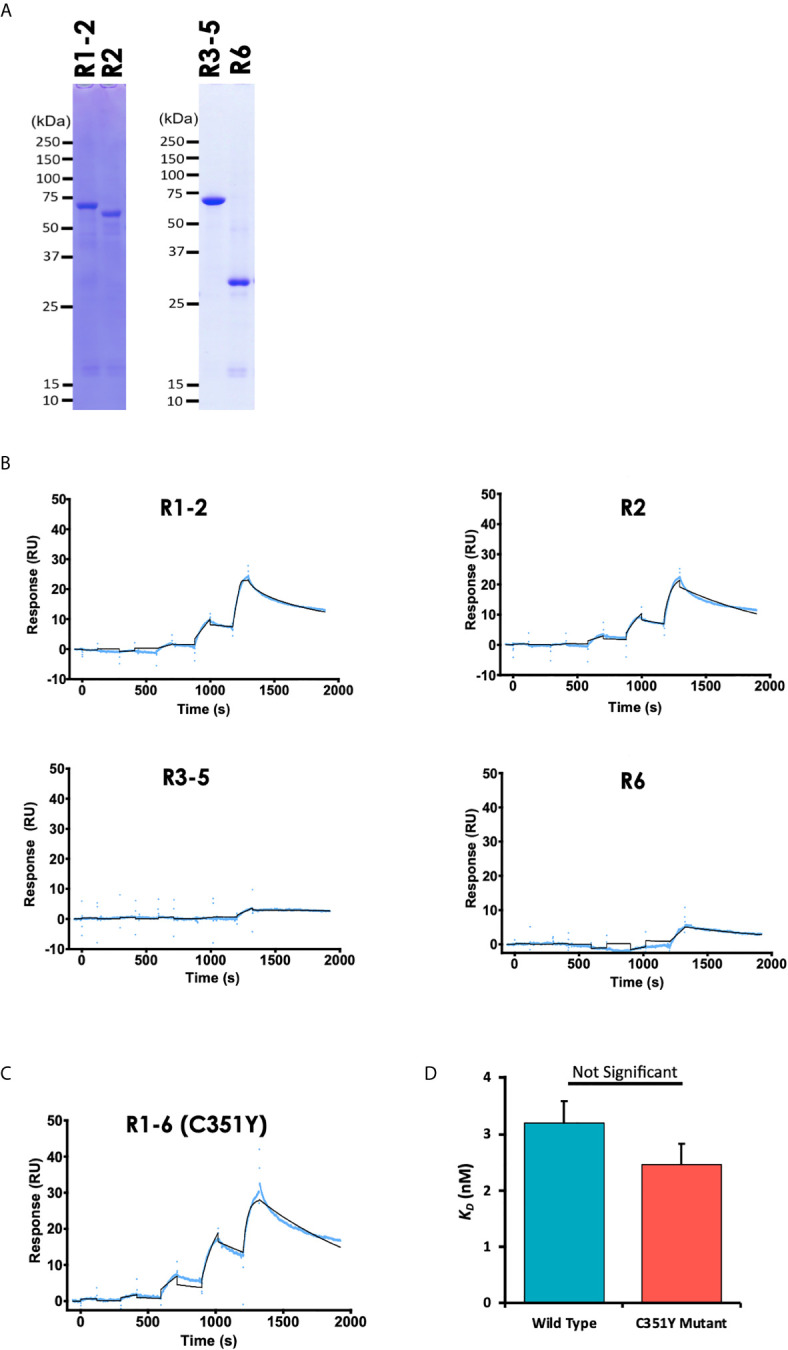
PyEBL interacts with basigin *via* the Region 2, the Duffy binding-like domain**. (A)** Recombinant PyEBL truncates namely R1-2, amino acid V_28_-G_437_; R2, E_113_-G_437_; R3-5, G_437_-C_717_; and R6, C_717_-N_788_ were expressed as N-terminal GST, C-terminal His-tagged proteins were purified with Ni-affinity columns, resolved by 12.5% SDS-PAGE under reducing conditions, and stained with CBB. Different truncates are shown. **(B)** Sensorgram of SPR single-cycle kinetic analysis between recombinant PyEBL truncates and recombinant mouse basigin. The SPR Chip used was same as used in **Figure 1D** with GST tagged Basigin as the analyte. The blue curve represents the actual data-generated sensorgram while the black curve indicates line of fit used to calculate kinetics parameters. R1-2 and R2 were assayed at an increasing protein concentration of 0.96, 4.8. 24, 120 and 600 nM. R3-5 and R6 were assayed at 62.5, 125, 250, 500 and 1000 nM at 120s and dissociation time of 180s. The last dissociation time was extended to 600s to accurately determine kinetic parameter. **(C)** Sensorgram of SPR single-cycle kinetic analysis between recombinant PyEBL C351Y and recombinant mouse basigin. The SPR Chip used was same as that used in **Figure 1D** with GST tagged basigin as the analyte. Blue curve represents the actual data-generated sensorgram while black curve indicates line of fit used to calculate kinetics parameters. The assay was performed at an increasing analyte concentration of 6, 12, 24, 48, and 96 nM at 120s and dissociation time of 180s. The last dissociation time was extended to 600s to accurately determine kinetic parameters. **(D)** Comparison of equilibrium binding constants between recombinant Basigin and recombinant PyEBL derived from *P. yoelii* wild type (PyEBL) or mutant (PyEBL C351Y). Each bar represents average SPR equilibrium binding constants of 4 independent experiments with error bars representing SE of the mean.

To determine the PyEBL region important for its interaction with erythrocyte surface protein basigin, we synthesized PyEBL R1-6 as truncated proteins with sections corresponding to the previously defined regions (Adams et al., 1992) namely region (R)1-2, amino acid V_28_-G_437_; R2, E_113_-G_437_; R3-5, G_437_-C_717_; and R6, C_717_-N_788_ (**Figures 1A**, **2A**). We observed that recombinant PyEBL R1-2 as well as R2, the N-terminal Cys-rich Duffy binding-like region (DBL) interacted with basigin at equilibrium binding constant (*K*_D_) of 1.6 ×10^-8^ M and 3.4 ×10^-8^ M, respectively (**Figure 2B**). The binding constants were comparable to that of PyEBL R1-6 (**Figure 1D**). In contrast, PyEBL R3-5 and PyEBL R6 did not bind to recombinant basigin (**Figure 2B**), suggesting that the region essential for interaction between PyEBL and basigin is located in R2.

Since a single amino acid substitution in PyEBL R6 domain determines the protein localization and influences the parasite’s virulence (Otsuki et al., 2009) while a mutation in the DBL domain affects both the parasite’s growth rate and virulence (Abkallo et al., 2017), we sought to determine whether the DBL C351Y mutation affects the ability of PyEBL to bind to its putative receptor. Sequences derived from the PyEBL C351Y mutant were cloned, confirmed by sequencing, and used to express recombinant protein. SPR analysis revealed slightly stronger albeit statistically non-significant difference in the binding kinetics between the C351Y mutant (*K*_D_ of 2.6 ×10^-8^ M) and wildtype derived R1-6 proteins (Student’s *t*-test, p = 0.23), suggesting the mutation had but a minimal effect on the protein’s binding capabilities to basigin (**Figures 2C, D**). Taken together, the data presented here suggest that PyEBL DBL (R2) is responsible for PyEBL interaction with basigin, and that this binding may not be affected by the C351Y mutation.

## Discussion

*Plasmodium* erythrocyte-binding-like proteins are involved in erythrocyte invasion by merozoites. In this study, by systematically screening a library of mouse erythrocyte proteins using PyEBL bait, we observed that the native parasite PyEBL protein interacts with basigin, the Ok blood group antigen. We further confirmed that region 2 of PyEBL is responsible for this interaction, consistent with the PyEBL orthologues of *P. falciparum* and *P. vivax* which are also known to interact with erythrocyte receptors through region 2 **(** Chitnis and Miller, 1994; Sim et al., 1994 **)**.

Basigin, also known as cluster of differentiation 147 (CD147) or extracellular matrix metalloproteinase inducer (EMMPRIN), is a type I integral membrane receptor member of the immunoglobulin superfamily with several distinct functions including spermatogenesis, expression of the monocarboxylate transporter and the responsiveness of lymphocytes. It has been implicated in the pathogenesis of several infectious and inflammatory diseases in which it has been extensively studied as a target of drug or vaccine based interventions (Muramatsu, 2012). Basigin has been evaluated as an essential receptor for *P. falciparum* Rh5 during human erythrocyte invasion (Crosnier et al., 2011). Antibodies against PfRh5 have a robust growth inhibitory activity making the molecule an attractive asexual blood-stage vaccine target (Healer et al., 2019). Here, we show that PyEBL interacts directly with mouse basigin using both native and purified recombinant proteins in AlphaScreen and SPR. Another protein, Band 3, was recently reported to co-immunoprecipitate with PyEBL (Peng et al., 2020). However, in our study, the protein did not show interaction with native PyEBL in the AlphaScreen assays probably due to the difference in the experimental approaches used. Put together, PyEBL-basigin interaction may act as an important key pathway that mediates attachment of merozoites to the erythrocyte surface, and this protein-protein interaction is an attractive model for vaccines and small molecule inhibitors studies.

Phenotypically, the *P. yoelii* nonlethal 17X parasite line mainly invades reticulocytes while the lethal lines 17XL and YM (carrying the C726R mutation) infect both reticulocytes and normocytes (Yoeli et al., 1975). This single amino acid substitution (C726R) in region 6, that harbors the intracellular trafficking domain (Culleton and Kaneko, 2010), alters the protein localization from the micronemes to the dense granules although the protein remains essential for parasite survival (Otsuki et al., 2009). It may also change the susceptibility of infected erythrocytes to complement pathways, and hence disease severity (Peng et al., 2020). Abkallo et al. observed that another single nucleotide polymorphism, C351Y, in the DBL domain (region 2) dramatically changed the parasite’s ability to invade erythrocytes and affected its growth rate (Abkallo et al., 2017) suggesting that the mutation influences the function of this gene. In addition, homology modeling suggested that the cysteine residue at position 351 forms a disulfide bond with the residue at position 420, and this bond is abolished following the C351Y substitution subsequently altering the DBL’ domain’s tertiary structure (Abkallo et al., 2017). In line with this model, we observed that C351Y mutant PyEBL bound basigin at a slightly lower affinity relative to the wild type. This could imply that the mutation might slightly strengthen the PyEBL interaction with basigin. In general, disulfide bonds enhance proteins thermodynamic stability making them more resistant to degradation as well as determining their tertiary structures and functional interactions (Liu et al., 2016). A C351Y substitution could increase flexibility of the PyEBL R2 *via* increased conformational freedom allowing it to not only bind to basigin, but also other uncharacterized receptor proteins. Nevertheless, further investigation would be required to elucidate the specific binding motifs as well as investigate if, and how the mutations expand the repertoire of erythrocyte receptors.

The findings presented here suggest that PyEBL R2 interacts with basigin. However, it remains unclear how the C726R polymorphism in PyEBL R6 enables mutant parasites to invade both reticulocytes and normocytes while still recognizing basigin. Further structural and functional studies on how R2 and R6 relates are required to elucidate these questions. Nonetheless, the identification of basigin as a putative PyEBL erythrocyte receptor offers new insights into the mechanism of invasion in malaria parasites.

## Data Availability Statement

The original contributions presented in the study are included in the article/**Supplementary Material**. Further inquiries can be directed to the corresponding authors.

## Ethics Statement

The animal study was reviewed and approved by Institutional Animal Care and Use Committee of Ehime University.

## Author Contributions

TT, HO, and ET conceived and designed experiments. TY, HN, TM, HT, and DI conducted experiments. BK, TT, and ET analyzed the data. BK, HO, TT, and ET wrote the manuscript. All authors contributed to the article and approved the submitted version.

## Funding

This work was supported in part by JSPS KAKENHI (Grant Nos. JP17H06873, JP18H02651, JP18K19455, JP19K22535, JP20H03481) and Takeda Science Foundation. The funders had no role in study design, data collection and analysis, decision to publish, or preparation of the manuscript.

## Conflict of Interest

The authors declare that the research was conducted in the absence of any commercial or financial relationships that could be construed as a potential conflict of interest.

## Abbreviations

EBL, Erythrocyte-binding-like; PBS, phosphate buffered saline; SDS-PAGE, sodium dodecyl sulfate-polyacrylamide gel electrophoresis.
