# Supplementary figures and images for: Plasmodium yoelii Erythrocyte Binding Like Protein Interacts With Basigin, an Erythrocyte Surface Protein
Source: Front Cell Infect Microbiol. 2021 Apr 14;11:656620. doi: 10.3389/fcimb.2021.656620 (PMC8079763; doi:10.3389/fcimb.2021.656620)

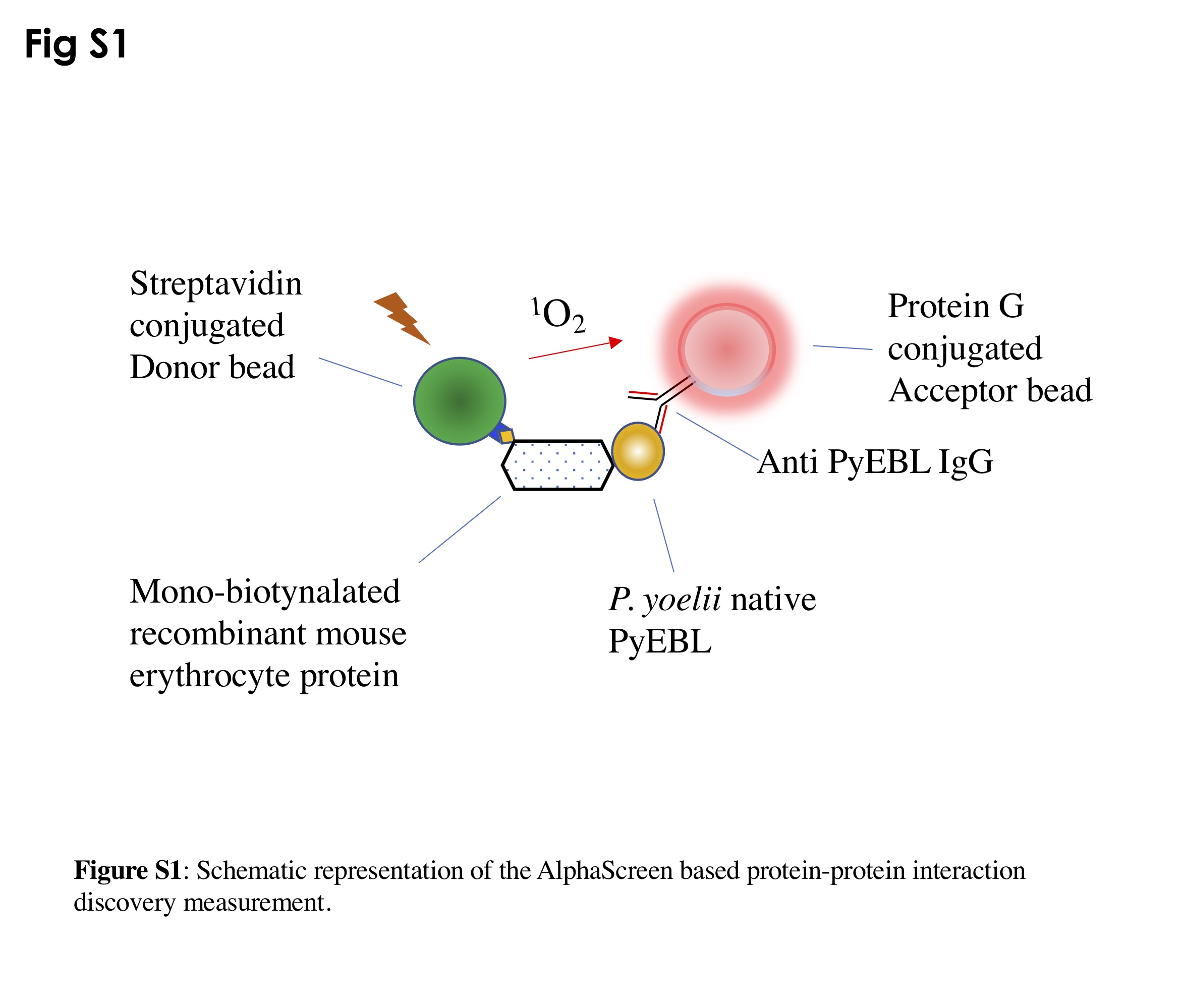

Supplement: Supplementary file 1 [file Image_1.jpeg]
