# Supplementary material for: Plasmodium yoelii Erythrocyte Binding Like Protein Interacts With Basigin, an Erythrocyte Surface Protein
Source: Front Cell Infect Microbiol. 2021 Apr 14;11:656620. doi: 10.3389/fcimb.2021.656620 (PMC8079763; doi:10.3389/fcimb.2021.656620)
Supplement: Supplementary file 2 [file Table_1.pdf]

Table S1: List of primers used in this study

**Name**	**Sequence** (5'->3')
PyEBL R1_R2_F	GAGAGACTCGAGATGGTTAATTTATTAAAAAGAACATATGAATCTTTCC
PyEBL R1_R2_R	TCTCTCGCGGCCGCCTAATGATGATGATGATGATGCAAATTATTATTAATAGGAGTATTACTGGGTTTAAC
PyEBL R2_F	GAGAGACTCGAGATGGAAAAAAATGGAAATGTAAATTACAAAGATTATTTTG
PyEBL R2_R	TCTCTCGCGGCCGCCTAATGATGATGATGATGATGCAAATTATTATTAATAGGAGTATTACTGGGTTTAAC
PyEBL R3_R5_F	GAGAGACTCGAGATGTCTTCTGTTAAACCCAGTAATACTC
PyEBL R3_R5_R	TCTCTCGCGGCCGCCTAATGATGATGATGATGATGTACATTTTCGTTGGCTAGCATTATTATA
PyEBL R6_F	ATCACTAGTTCTCGAGATGTGCAATGGCAATCCAGGTTTAAAA
PyEBL R6_R	TCTCTCGCGGCCGCCTAATGATGATGATGATGATGTGAATAGCTCTCTTTTTGAAAACAG
PyEBL R1_R6_F	GAGAGACTCGAGATGGTTAATTTATTAAAAAGAACATATGAATCTTTCC
PyEBL R1_R6_R	TCTCTCGCGGCCGCCTAATGATGATGATGATGATGTGAATAGCTCTCTTTTTGAAAACAG
PyEBLR1_R6 (C351Y)_F	AATGCGTACAATACATATGAGGCATGGATAAGTGT
PyEBLR1_R6 (C351Y)_R	TGTATTGTACGCATTTTTACATTCATTATTAATACATATACTTT
Basigin_F	GAGAGAGACTCGAGATGGCGGGCACCATCCAAAC
Basigin_R	TCTCTCTCGCGGCCGCTCAATGATGATGATGATGATGCATGCGGCTCCGCAC
