# Supplementary material for: Plasmodium yoelii Erythrocyte Binding Like Protein Interacts With Basigin, an Erythrocyte Surface Protein
Source: Front Cell Infect Microbiol. 2021 Apr 14;11:656620. doi: 10.3389/fcimb.2021.656620 (PMC8079763; doi:10.3389/fcimb.2021.656620)
Supplement: Supplementary file 6 [file Table_5.pdf]

Table S5: Kinetics Parameters determined by the SPR analysis

Name	ka (1/Ms)	kd (1/s)	KD (M)	U-value
**His-GST**	N/A	N/A	N/A	N/A
**R1-6**	3.2×10^{4}	1.0×10^{-3}	3.3×10^{-8}	2
**R1-2**	4.4×10^{5}	7.1×10^{-3}	1.6×10^{-8}	15
**R2**	3.1×10^{4}	1.0×10^{-3}	3.4×10^{-8}	2
**R3-5**	N/A	N/A	N/A	N/A
**R6**	N/A	N/A	N/A	N/A
**R1-6 (C351Y)**	3.7×10^{4}	9.7×10^{-4}	2.6×10^{-8}	3
